# Repeated stereotactic radiosurgery for high grade meningioma

**DOI:** 10.1007/s11060-025-05165-z

**Published:** 2025-08-13

**Authors:** Tzu-Chiang Peng, Ming-Hsuan Hsieh, Chun-Fu Lin, Ai Seon Kuan, Cheng-Chia Lee, Hsiu-Mei Wu, I-Chun Lai, Huai-Che Yang

**Affiliations:** 1 Department of Neurosurgery, Neurological Institute, Taipei Veterans General Hospital, Taipei, Taiwan; 2 School of Medicine, National Yang Ming Chiao Tung University, Taipei, Taiwan; 3 Institute of Public Health, College of Medicine, National Yang Ming Chiao Tung University, Taipei, Taiwan; 4 School of Medicine, College of Medicine, National Yang Ming Chiao Tung University, Taipei, Taiwan; 5 Department of Radiology, Taipei Veterans General Hospital, Taipei, Taiwan; 6 Department of Heavy Particles & Radiation Oncology, Taipei Veterans General Hospital, Taipei, Taiwan; 7 School of Medicine, National Defense Medical Center, Taipei, Taiwan

**Keywords:** Atypical meningioma, Anaplastic meningioma, Stereotactic radiosurgery, Recurrence, Repeated radiosurgery, Complication

## Abstract

**Background and objectives:**

Atypical and anaplastic meningioma (World Health organization [WHO] Grades II and III) present therapeutic challenges due to their aggressive behavior and high risk of recurrence. This study addressed the lack of data related to the effectiveness and safety of repeated stereotactic radiosurgery (SRS) in managing high-grade recurrent or residual meningioma.

**Methods:**

This study analyzed data extracted from the medical records of 112 patients (mean age of 57.9 years) who underwent SRS for recurrent or residual high-grade meningioma between January 2002 and December 2022. The data included clinicodemographic profiles, treatment parameters, and imaging phenotypes. The average follow-up duration was 41.7 months (range 12-160.7 months).

**Results:**

There was slight preponderance of females in the cohort (65:47). All patients had undergone craniotomy and histopathologic confirmation of atypical or anaplastic meningioma. Gross tumor resection was achieved in 35 cases. Atypical meningioma accounted for the vast majority of cases (105; 93.8%) with only 7 cases of anaplastic meningioma (6.2%). At a median follow-up of 41.7 months after SRS, tumor control was achieved in 29.5% of cases. Kaplan-Meier analysis indicated overall actuarial progression free survival rates of 79.5%, 43.8% and 30.4% at 1, 3 and 5 years after radiosurgery, respectively. In cases involving repeated SRS, these rates were 97.6%, 69% and 45.2%, respectively. Male sex, older age, anaplastic phenotype, subtotal resection, and larger tumor volume were significantly predictive of tumor growth after treatment. Adverse radiation effects were observed in 33.8% of patients who underwent repeated SRS. Most of those patients were asymptomatic and only five patients required a temporary course of steroid therapy.

**Conclusions:**

Our data suggest that SRS is a relatively safe and effective treatment option for recurrent or residual high-grade meningioma, with an acceptable complication profile, even when performed repeatedly. Anaplastic tumor phenotype, subtotal resection, and larger tumor volume were factors associated with tumor growth, warranting close clinical surveillance after radiosurgery.

## Introduction

Meningioma is the most common intracranial tumor, accounting for 25–33% of all brain tumors [1, 2]. The World Health Organization (WHO) classifies meningioma into three categories according to histopathological features: benign (Grade I), atypical (Grade II), and anaplastic (Grade III) [3]. Atypical and anaplastic meningioma respectively account for roughly 5% and 1.0% of all meningiomas. These high-grade tumors present a higher mitotic count, necrosis, sheath-like growth pattern, compact cellularity, and prominent nucleoli [4]. They also tend to behave aggressively and are associated with a poor prognosis [5, 6].

Meningioma treatment generally focuses on maximal safe resection, as the extent of resection strongly correlates with tumor recurrence [7, 8]. In a study on atypical meningioma, Modha et al. reported a 17% recurrence rate for gross total resection, compared to 87% for subtotal resection [4]. Other researchers have reported similar findings [9]. Although most neurosurgeons seek to achieve resection to the fullest extent, doing so while preserving neurological function can be challenging, particularly in eloquent or deep-seated locations.

Radiotherapy is widely used as an adjuvant therapy for recurrent or residual high-grade meningioma [10–12]. Stereotactic radiosurgery (SRS) is a minimally invasive alternative, providing conformal, highly focused, single-fraction irradiation with steep dose fall-off, sparing adjacent normal tissue. Early studies have reported that when treating atypical or anaplastic meningioma, the outcomes SRS is comparable to that of traditional radiotherapy [13, 14]. However, there is a lack of data pertaining to the best adjuvant radiotherapy course for high-grade meningioma. This study assessed the effectiveness and safety of repeated SRS in the management of high-grade recurrent or residual meningioma.

## Methods

Data were collected from a single academic medical center between January 2002 to December 2022, following approval by the institutional review board.

Inclusion criteria were as follows: (1) histopathological confirmation of atypical or anaplastic meningioma; (2) availability of sufficient data on basic clinicodemographic characteristics, SRS treatment parameters, and imaging features; (3) no exposure to radiation prior to the current radiosurgery; (4) a minimum 12 months of imaging and clinic follow-up.

### Baseline data and variables

Basic clinicodemographic profiles included age, sex, surgical outcomes and the interval between operation and radiosurgery. Extent of tumor resection was defined based on intraoperative findings as either gross tumor removal (Simpson grade 1–2) or subtotal resection (Simpson grade 3–5). Tumor variables included meningioma location, volume, WHO grade, and indication of SRS.

### Follow-up evaluation

Patients were discharged on the day of the SRS procedure or the day after. Post-SRS follow-up included imaging studies and brain MRI at 3-month intervals. The tumor volume was calculated primarily via the a*b*c/2 approach. To avoid subjective variation in imaging interpretation, a cutoff of 10% change in tumor volume was set. Tumor progression was defined as a > 10% increase in the volume of a pre-existing lesion or the appearance of a new marginal tumor. Tumors that remained within 10% of their volume at the time of radiosurgery were classified as stable. For those demonstrating infield tumor progression or outfield recurrence, repeated SRS was suggested in the absence of symptomatic mass effect.

Radiation induced changes (RICs) were defined as new-onset or increased peritumoral hyperintensity on a T2-weighted MRI scan in the absence of tumor progression. Symptomatic RICs implied that the worsening of an ailment or newly-developed neurological deficits were attributable to these changes. Time to RIC onset was defined as the interval between the date of SRS and the radiographic detection of edema.

### Radiosurgical methodology

In the current study, 3 patients were treated using Gamma Knife model B, 29 patients using Gamma knife model 4 C and 80 patients using Gamma knife Perfexion. Following stereotactic frame placement, patients underwent thin-slice magnetic resonance imaging (MRI) with intravenous contrast media. Tumor delineation, dose selection, and planning were performed by a neurosurgeon in conjunction with a radiation physician. In most cases, the meningiomas were well-enhanced, showing a sharp demarcation with peripheral structures. The planning target volume was defined as the gross tumor volume with no additional margin. All patients received single-fraction SRS and none had hypo-fractionation radiosurgery.

### Statistical analysis

Data are presented as mean, median, and range for continuous variables and percentages for categorical variables. Normality was assessed graphically and statistically. Statistical analysis for continuous variables was performed using an unpaired Student t-test. In cases where continuous variables were not normally distributed, the Wilcoxon test was applied. For categorical variables, Pearson‘s chi-squared test was performed as appropriate. A statistically significant difference was considered as *p* < 0.05.

Clinicodemographic variables, tumor characteristics, and treatment parameters were assessed using multivariable Cox proportional hazards models to identify predictors of tumor recurrence. Factors with *p* < 0.05 was considered statistically significant. To account for the long study interval with variability in imaging, surgical technique and radiosurgical platform, we did sensitivity analyses by running a stratified Cox model stratifying the patients by their radiosurgical platform: Gamma Knife model B (year 1993–2006), Gamma Knife model 4 C (year 2007–2013), and Gamma Knife Perfexion (year 2014 onwards).

## Results

### Demographic variables and baseline characteristics

**Fig. 1:**
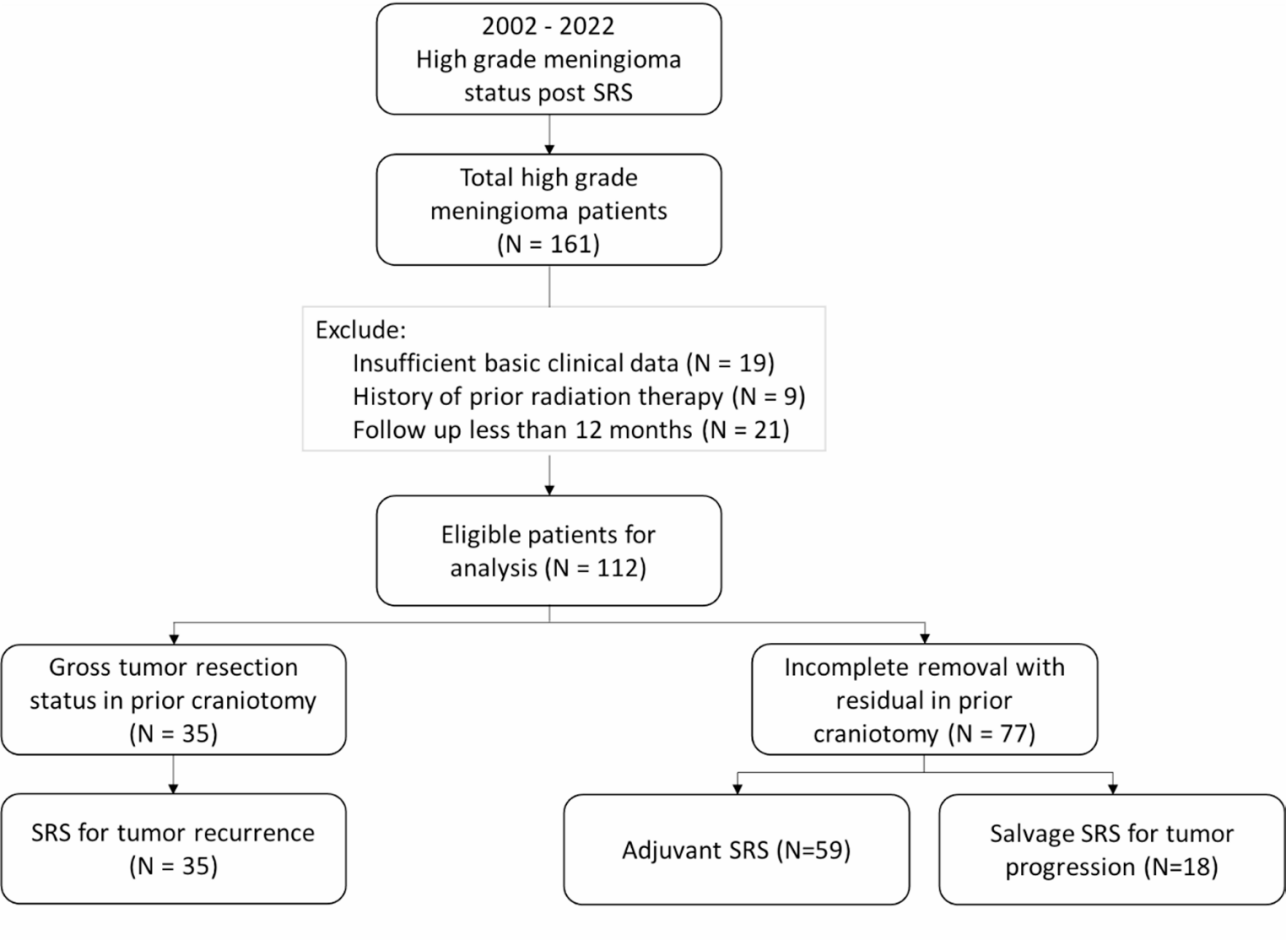
Flowchart showing the process of selecting patients who underwent SRS for atypical/anaplastic meningioma SRS = stereotactic radiosurgery

Figure 1 presents a flow chart showing the patient selection process. Between January 2002 and December 2022, 161 patients underwent radiosurgery for atypical or anaplastic meningioma. Patients were excluded if they had undergone craniotomy in another institution, such that histopathology results could not be confirmed (*n* = 19), had undergone radiotherapy prior to SRS (*n* = 9), or had inadequate follow-up (*n* = 21). After applying these criteria, 112 cases were deemed eligible for evaluation.

Table 1 summarizes the baseline patient and tumor characteristics. The study cohort presented a slight preponderance of females (65:47) and a mean age of 57.9 years old. Gross tumor removal was achieved in 35 subjects and subtotal resection in 77 cases. The mean elapsed time between the date of craniotomy and radiosurgery was 14.4 months.

SRS was performed as adjuvant therapy for residual tumors in 59 patients. In the remaining 53 patients, SRS was used as salvage therapy—either for tumor progression (*n* = 18) or recurrence (*n* = 35). Atypical meningioma accounted the majority of cases (105; 93.8%) with a mean volume of 14.9 ml. The most common meningioma locations were the skull base (41 cases; 36.6%), parasagittal region (35 cases; 31.3%), and convexity (16 cases; 14.3%).

**Table 1:** Summary of high-grade meningioma treated by SRS

Characteristics		Mean/median	Range
Age		57.9/59.4	21.9–89.7
Sex			
	Male	47 (42%)	
	Female	65 (58%)	
Tumor location			
	Base	41 (36.6%)	
	Parasagittal	35 (31.3%)	
	Convexity	16 (14.3%)	
	Falx	8 (7.1%)	
	Tentorium	4 (3.6%)	
	CPA	4 (3.6%)	
	Ventricle	4 (3.6%)	
Pathological grade			
	Atypical	105 (93.8%)	
	Anaplastic	7 (6.2%)	
Resection status			
	STR	77 (68.7%)	
	GTR	35 (31.3%)	
Interval between last craniotomy and SRS		14.4/6.6	0.6-125.4
SRS indication			
	Residual tumor	59 (52.6%)	
	Tumor progression	18 (16.1%)	
	Tumor recurrence	35 (31.3%)	
Radiosurgery parameter			
	Target volume(cm^3^)	13.8/10	0.4–73.6
	Maximal dose	25.9/25.5	18.2–40
	Marginal dose	14.7/15	11.9–20
SRS sessions			
	One	70 (62.5%)	
	Two	25 (22.3%)	
	Three	9 (8%)	
	Four	7 (6.3%)	
	Five	1 (0.9%)	

SRS = stereotactic radiosurgery; CPA = cerebellopontine angle; STR = subtotal tumor resection; GTR = gross tumor resection

### Post-operative follow-up and tumor recurrence

Table 2 details the outcomes of SRS in managing high-grade meningioma. The mean marginal and maximal SRS doses were 14.7 and 25.9 Gy, respectively. The mean follow-up period was 41.7 months.

A total of 79 patients experienced treatment failure, either as infield tumor progression (*n* = 48) or outfield recurrent tumor (*n* = 31), with a mean time to progression of 24.4 months following SRS. Two patients died due to uncontrollable tumor growth with profound neurological impairment at 50.4 and 56.2 months postoperatively.

Among the 79 subjects with tumor progression, 42 underwent repeated radiosurgery, 24 underwent surgical resection, and 1 opted for boron neutron capture therapy. Kaplan–Meier plots revealed progression free survival (PFS) of 79.5% at 1 year, 43.8% at 3 years, and 30.4% at 3 years.

**Table 2:** Outcomes of radiosurgery

Outcomes	Mean/median	Range
Infield progression	48 (42.9%)	
Outfield recurrence	31 (27.7%)	
Stable or regression	33 (29.5%)	
Time to tumor progression	22.4/19.7	3.4–67.1
RIC	23 (20.5%)	
Interval between SRS and RIC in months	6.1/5	2.1–18.3
Follow up duration in months	41.7/31.7	4.8-160.7
End point		
Still follow up	45 (40.2%)	
Craniotomy	24 (21.4%)	
Repeat SRS	42 (37.5%)	
BNCT	1 (0.9%)	

ARE = adverse radiation effect; RIC = radiation induced change; SRS = stereotactic radiosurgery; BNCT = Boron Neutron Capture Treatment

### Predictors of tumor recurrence

Table 3 outlines the results of multivariate Cox proportional hazard analyses evaluating factors associated with tumor progression following radiosurgery for high-grade meningioma. Male sex, older age, anaplastic tumor phenotype, subtotal resection, and larger tumor volume were significantly predictive of tumor growth. Sensitivity analyses accounting for variability in imaging, surgical techniques, and radiosurgical platform over the long study period showed results similar to Table 3 (results not shown).

**Table 3:** Predictors of high-grade meningioma progression

	Multivariate Analysis		
Characteristic	OR	95% CI	*p* value
Male sex	2.06	1.17–3.62	**0.012**
Age	1.02	1.00-1.04	**0.031**
WHO grade 3	2.90	1.18–7.09	**0.020**
Skull base location	0.83	0.50–1.37	0.472
Subtotal resection	0.55	0.32–0.96	**0.035**
Salvage therapy	1.30	0.73–2.30	0.371
Tumor volume ≥ 10.2 ml *	2.01	1.18–3.41	**0.010**
Margin dose ≥ 14 *	1.33	0.67–2.64	0.409
Maximal dose ≥ 25 *	0.74	0.40–1.37	0.335

*The cutoff values for tumor volume, margin dose and maximal dose were based on the median values of the study participants

### Toxicity of radiosurgery

Post-treatment brain edema was observed in 23 patients following their initial SRS session. The mean elapsed time between SRS and RICs detection was 6.1 months. Most patients were asymptomatic, such that the adverse effects were detected incidentally during routine imaging follow-up. Five patients reported transient symptoms—increased in seizure frequency (*n* = 3), headache(*n* = 2), and/or mild muscle weakness (*n* = 1)—which were managed with a temporary course of steroids and had complete reversal of symptoms. None of the patients in our study cohort reported longstanding neurological consequences in the absence of tumor progression.

### Repeated SRS and Progression-Free survival (PFS)

In our study cohort, 42 subjects underwent repeated radiosurgery for progressive or recurrent high-grade meningioma. This group was further subdivided according to the number of SRS sessions they received, as follows: two sessions (*n* = 25), three sessions (*n* = 9), four sessions (*n* = 7), and five sessions (*n* = 1). These groups were compared with single session groups in terms of baseline characteristics, treatment parameters, and clinical outcomes (Table 4).

**Fig. 2:**
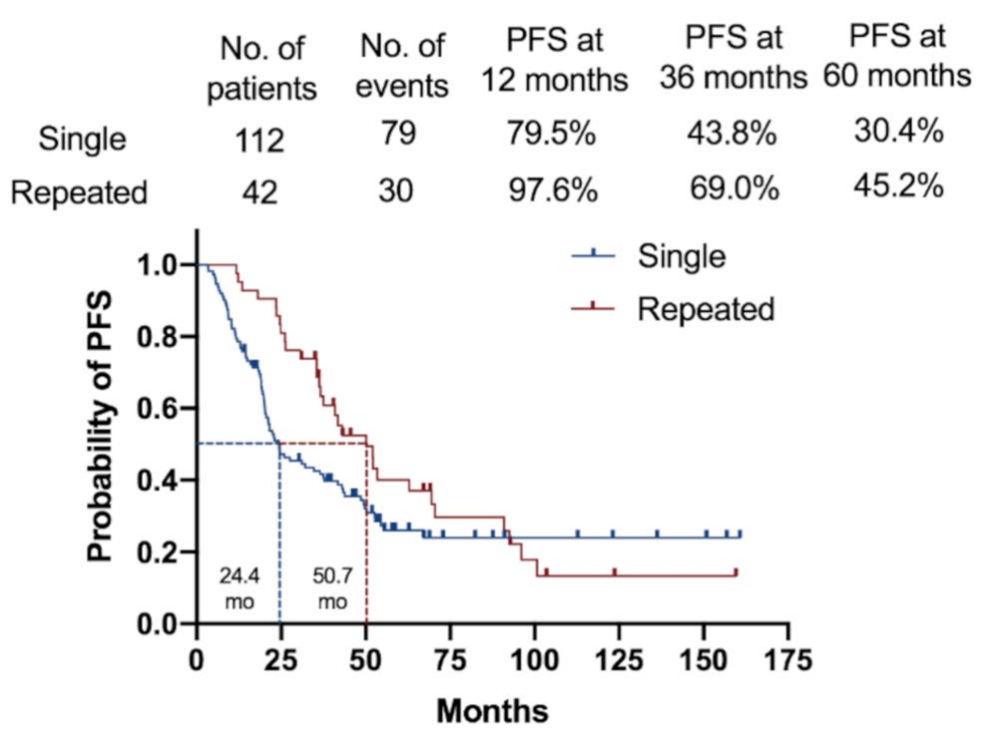
Kaplan–Meier curves showing tumor control rates following stereotactic radiosurgery (SRS) for high-grade meningioma. The lower blue line indicates the tumor control rate after a single SRS session, whereas the red line indicates the outcomes following repeated SRS for recurrent tumors. Tumor control rates after repeated SRS were 97.6%, 69%, and 45.2% at 1, 3, and 5 years, respectively PFS = progression free survival; SRS = stereotactic radiosurgery

Following repeated SRS, the actuarial progression free survival rate increased to 97.6%, 69%, and 45.2% at 1, 3, and 5 years, respectively. Additionally, the mean time to tumor progression increased significantly from 24.4 to 50.7 months (Fig. 2).

Stratified by the pattern of treatment failure, there were 31 patients received repeated radiosurgery for infield tumor progression and 37 patients sustained outfield recurrent tumor. Clinicodemographic profile, treatment parameter and radiation outcomes were compared in Table 5. It was noticed that radiation volume of infield group was larger than the outfield group (*p* = 0.006), but there was no significant difference in the rate of ARE occurrence or tumor progression.

**Fig. 3:**
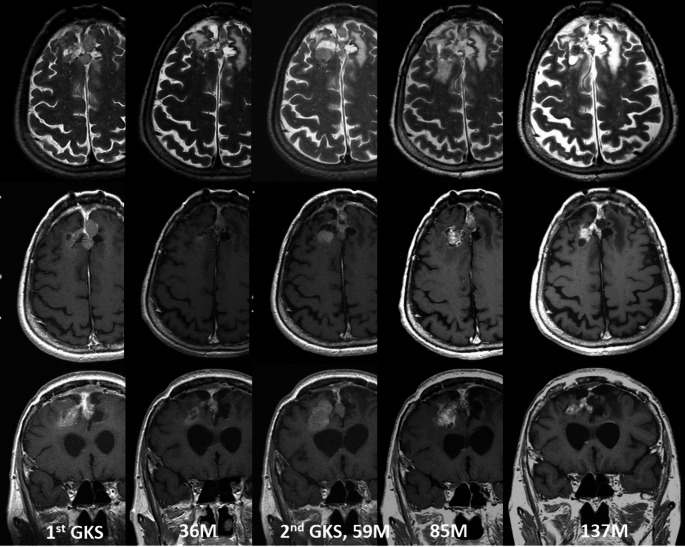
A 47-year-old female patient presented frontal falx atypical meningioma (10 cm^3^) post-craniotomy. The patient initially underwent SRS at a marginal dose of 12.4 Gy. Follow-up imaging revealed a significant decrease in the volume of the tumor; however, this was followed by tumor recurrence 59 months later. The patient underwent a second SRS session at a marginal dose of 13.6 Gy. Over the subsequent 6 years, the tumor volume continued to regress, with the patient remaining neurologically intact throughout the course of treatment

We also compared single and multi-session cases in terms of RICs incidence. RICs was observed in 23 cases with a mean onset interval of 5.2 months. No significant difference was observed between the two groups (relative risk 1.65, *p* = 0.058). Figure 3 presents an illustrative case of a 47-year-old female patient with residual atypical meningioma who underwent two sessions of SRS, achieving effective tumor control.

**Table 4:** Comparison of patients who underwent single or repeated SRS sessions for high-grade meningioma

SRS session	Single (*n* = 112)	Repeated (*n* = 68)	*p* value
Female: male	65:47	38:30	0.779
Mean age	59.9	63.4	**0.010**
Skull base: non-base	45:67	29:39	0.286
Atypical: anaplastic	105:7	66:2	0.326
Target volume in cm^3^	14.9	12.1	0.178
Maximal dose in Gy	25.4	26.8	**0.010**
Marginal dose in Gy	14.5	15.1	**0.021**
ARE	23	23	0.058
Mean time to RICs in months	6.1	5.2	0.560
Mean time to tumor progression in months	31.1	70.6	**< 0.001**
One-year PFS	79.5%	97.6%	
Three-years PFS	43.8%	69.0%	
Five-year PFS	30.4%	45.2%	

SRS = stereotactic radiosurgery; PFS = progression free survival

**Table 5:** Comparison of patients who underwent repeated SRS for infield or outfield high-grade meningioma

Pattern of recurrent meningioma	Infield (*n* = 31)	Outfield (*n* = 37)	*p* value
Female: male	18:13	19:18	0.586
Mean age	65.7	61.2	0.126
Target volume in cm3	16	8.3	**0.006**
Maximal dose in Gy	26.4	27	0.503
Marginal dose in Gy	14.9	15.2	0.365
ARE	13	9	0.102
Mean time to RICs in months	6.1	4.2	0.401
Tumor progression	25	29	0.821
Mean time to tumor progression in months	12.3	13.8	0.611

## Discussion

Atypical/anaplastic meningioma are known for higher recurrence rates and poorer survival rates, compared to their benign counterparts [5]. Early literature on the management of high-grade meningioma focused on maximizing safe resection through conventional radiotherapy [11, 15, 16]. Those studies reported dismal overall outcomes, with 5-years PFS rates between 32% and 48%, and 5-year overall survival rates between 28% and 95% [17–21].

In a retrospective review of patient databases, Klinger et al. identified a higher MIB-1 labeling index, higher mitotic figure, and subtotal resection as significant predictors of tumor recurrence involving non-skull base meningioma [22]. Their findings suggest that adjuvant radiotherapy alone may be insufficient for long-term tumor control, necessitating other treatment modalities.

### SRS for high-grade meningioma

SRS has emerged as a minimally invasive approach for the management of residual or recurrent meningioma, enabling the conformal, highly focused, single fraction irradiation of irregular tumor volumes with steep dose fall-off. In their use of radiosurgery to treat residual/recurrent atypical meningioma, Clara et al. achieved outcomes comparable to those of conventional radiotherapy, with overall locoregional control rates of 93%, 93% and 54% at 12, 24 and 36 months post-surgery [13].

Based on a series of 228 patients who underwent microsurgical procedures for atypical meningiomas, Hardesty et al. reported that immediate postoperative adjuvant SRS did not significantly impact tumor recurrence rates, regardless of the extent of resection [23]. They advocated an expectant approach, such that aggressive surgical resection and irradiation are considered only at the time of tumor progression. Nonetheless, the issue of selecting an optimal adjuvant treatment regimen remains an issue of debate, with considerable variation in practice patterns across institutions.

In the current study, 59 patients underwent immediate adjuvant radiosurgery and 53 underwent salvage therapy for progressive (*n* = 18) or recurrent tumor (*n* = 35). For the former 59 cases of subtotal resection status, the planning target volume of SRS was defined as the residual tumor volume but not whole tumor bed. No significant difference was observed between adjuvant radiosurgery and salvage therapy (*p* = 0.371). These findings support the viability of an expectant approach with close radiographic monitoring as a safe and reasonable strategy for patients with small residual high-grade meningiomas when radiosurgery is viable.

Due to the inherent malignancy of high-grade meningioma, many surgeons face a dilemma in dealing with tumors involving incomplete resection or recurrence. The ability to identify aggressive meningioma could be beneficial in guiding treatment decisions. In our series, incomplete tumor resection was one of the variable that was predictive of tumor recurrence after radiosurgery. Numerous theories have been posited to explain the mechanism underlying this finding. One theory is that post-operative changes in the imaging study could potentially interfere tumor delineation and planning, resulting in inadequate tumor coverage [23–25]. Bin et al. reported that the tumor control rates and complication profiles of primary SRS were superior to those of adjuvant SRS for petroclival meningioma [25]. In another comprehensive study, Kaur et al. performed systemic analysis aimed at elucidating the effects of adjuvant radiotherapy in treating high-grade meningioma. In that study, incomplete resection and lower radiation doses were associated with inferior tumor control rate [10]. These findings support the viability of an expectant approach with close radiographic monitoring as a safe and reasonable strategy for patients with small residual high-grade meningioma when SRS is applicable [4, 9].

### Efficacy and safety of repeated radiosurgery

Among cases of high-grade meningioma, marginal or distal tumor recurrence after initial SRS is common [24]. Despite the extensive use of repeated SRS in clinical practice, there is a lack of data on long-term outcomes. Moreover, treatment strategies are largely extrapolated from data on benign meningioma, resulting in nonuniform protocols across institutions.

In our series, locoregional control rates and complication profiles did not vary considerably between those who underwent one SRS and those who underwent multiple sessions; however, implementing multiple SRS sessions increased the time to tumor progression from 24.4 to 50.7 months. In complication analysis, we found no significant difference in the rate of ARE for single or repeated SRS. Although, infield high grade meningioma was larger than the outfield counterparts in our cohort, it did not translate into lower radiation response rate or untoward side effects.

Our results support the feasibility and safety of repeated SRS as a feasible and safe treatment modality for recurrent or progressive high-grade meningioma, offering durable tumor control without a significant increase in toxicity.

### Limitations

This study was subject to various limitations, which should be considered in the interpretation of the findings. As a retrospective study, this work was inherently subject to selection bias based on practitioner experience, and the single tertiary referral center design limited the generalizability of our findings. The heterogeneity of available data also posed challenges to the consistency and comparability of certain variables. Although this series was one of the largest series dealing with issue, large-scale, prospective studies with well-defined patient selection criteria are required to validate and expand upon our findings.

## Conclusions

Stereotactic radiosurgery is emerging as a potentially effective and minimally invasive modality for the management of high-grade meningioma; however, the optimal treatment paradigm and therapeutic outcomes have yet to be fully elucidated. Based on our front-line experience, this study supports the use of repeated SRS as a viable and safe strategy for managing recurrent or residual high-grade meningiomas, with favorable tumor control and acceptable toxicity profiles.

Our data also suggest that male sex, older age, anaplastic tumor phenotype, subtotal resection, and larger tumor volume were significantly predictive of tumor growth after treatment. It is imperative to perform close clinical follow-up in these cases.

## Data Availability

No datasets were generated or analysed during the current study.
